# Sub-Chronic Difenoconazole Exposure Induced Gut Microbiota Dysbiosis in Mice

**DOI:** 10.3390/toxics10010034

**Published:** 2022-01-12

**Authors:** Zhiwei Bao, Weitao Wang, Xiaofang Wang, Mingrong Qian, Yuanxiang Jin

**Affiliations:** 1 Department of Biotechnology, College of Biotechnology and Bioengineering, Zhejiang University of Technology, Hangzhou 310032, China; 2111805009@zjut.edu.cn (Z.B.); 2112005014@zjtu.edu.cn (W.W.); 2112005012@zjut.edu.cn (X.W.); 2 Environmental Chemistry and Expourse Research, Interdisciplinary Research Academy, Zhejiang Shuren University, Hangzhou 310032, China

**Keywords:** fungicides, Difenoconazole, gut microbiota, mice

## Abstract

Difenoconazole (DIF) is a widely separated triazole fungicide in many countries. The excessive usage of DIF increases the high volume of residues in agriculture production and water bodies. Some previous studies demonstrated the toxic effects of DIF on non-target animals, however, there were still some gaps in the knowledge of the potential hazards of DIF to mammals and human health. Herein, 7-week-old male mice were exposed to 30 and 100 mg/kg/day DIF for 14 and 56 days. We observed that 56 days of DIF exposure decreased the colonic mucus expression of alcin blue-periodic acid-schiff (AB-PAS) stain and the immunochemical stain of *muc2* protein. The transcript levels of mucin protein (*muc1*, *muc2* and *muc3*) decreased significantly in the gut of mice followed 56 days of 100 mg/kg/day DIF exposure. In addition, the gut microbiota composition was also affected after 14 or 56 days of DIF exposure. Although the mucus expression after 14 days of DIF exposure only decreased slightly, the gut microbiota composition compared with the control group was changed significantly. Moreover, the DIF-30 and DIF-100 caused respectively different changes on the gut microbiota. The relative abundance of Bacteroidetes decreased significantly after 14 days and 56 days of DIF exposure. After 14 days of DIF exposure, there were 35 and 18 differential genera in the DIF-30 and DIF-100 group, respectively. There were 25 and 32 differential genera in the DIF-30 and DIF-100 group after 56 days of exposure, respectively. Meanwhile, the alpha diversity indexes, including observed species, Shannon, Simpson, Chao1 and ACE, in gut microbiota decreased significantly after 56 days of DIF exposure. Interestingly, the relative abundance of *Akkermansia* increased significantly after 56 days of 100 mg/kg/d DIF exposure. Although *Akkermansia* was considered as one probiotic, the phenomenon of dramatic *Akkermansia* increase with the decrease in gut microbiota diversity needed further discussion. These results provided some new insights on how DIF exposure impacts the mucus barrier and induces gut microbiota dysbiosis.

## 1. Introduction

Triazole fungicides can effectively control pathogen fungus by inhibiting the C14-demethylase in sterol synthesis. Sterols, especially ergosterol, play important roles in the cell membrane formation. The lack of sterols inhibited by triazole fungicides causes abnormal fungal growth, even death. Difenoconazole (DIF) is an internal absorption triazole fungicide. Due to its highly efficient and broad spectrum, DIF is widely used worldwide to combat fungal diseases [1].

With the wide usage of DIF, DIF residue was frequently detected in agriculture production. For example, Cui et al. showed that DIF was frequently detected in a total of 3406 fruit and vegetable samples, especially in cowpea (60.30%, maximum 343.39 μg/kg), grape (68.70%, maximum 803.30 μg/kg) and mandarin (64.34%, maximum 192.70 μg/kg) [2]. DIF was also detected in 43% of tomato crops (maximum 64.3 μg/kg) in Western Algeria [3]. DIF was not only detected in agriculture productions, but also in the water bodies due to its high persistence [4,5,6]. Because it widely exists in the environment and food, the toxicity of IDF was analyzed by some groups. For instance, some previous studies demonstrated that DIF exposure caused toxic effects in non-target organisms, such as zebrafish [7,8], marine medaka [9], honey bee [10]. More recently, Wang et al. demonstrated that 80 μM DIF exposure could induce reactive oxygen species (ROS) generation and cause DNA damage in the human neuroblastoma SH-SY5Y [11]. Wang et al. (2021a) also reported that 30 μM DIF exposure induced cytotoxicity and apoptosis via ROS in HepG2 cells [12]. However, there was a lack of knowledge of the effects of DIF on mammals.

As we know, in animals, the gut is considered to be the first line of interaction with xenobiotics exposure [13]. To support this idea, a number of previous studies proved that xenobiotics exposure could cause gut microbiota dysbiosis and impact the intestinal barrier and even affect the host health [14,15]. The mucus layer, one crucial part of intestinal barrier, has multiple functions as a physical and immunological defense barrier [16]. There are numerous microbiotas living in the colon and the mucus layer could prevent invasion [16]. When the mucus layer is defective, bacteria invade and cause an immune response [17]. Our previous studies demonstrated that pesticide exposure affects gut microbiota composition, mucus secretion and impacts gut barrier [18,19]. However, whether DIF could affect the gut microbiota and barrier function in mice remained unclear. Previous study showed that the no observed adverse effect level (NOAEL) of DIF in rats after 21 d chronic exposure was >189 mg/kg BW/d, thus, we chose the100 mg/kg BW/d (under NOAEL) as maximum exposure concentration. Herein, 7-week-old male mice were given a gavage with 30 mg/kg BW/d and 100 mg/kg BW/d DIF for 14 days and 56 days. After exposure, the colonic mucus layer and gut microbiota were investigated. These results acquired could provide new insights and raise concern about gut health and DIF exposure. 

## 2. Methods and Materials

### 2.1. Chemicals and Experimental Animal Husbandry

Difenoconazole (CAS No. 119446-68-3) was purchased from Dr. Ehrenstorfer GmbH (Augsburg, Germany). A total of 48 6-week-old male C57BL/6 mice were purchased from the China National Laboratory Animal Resource Center (Shanghai, China). All mice were kept in an animal facility with preference temperature (22 ± 2 °C) and 12 h light/12 h dark photoperiod. After one week acclimation, the mice were randomly selected into 3 groups (*n* = 16): (1) control group (Con), (2) 30 mg/kg/d DIF-treated group (DIF-30) and (3) 100 mg/kg/d DIF-treated group (DIF-100). DIF was dissolved in corn oil (Aladdin, China) and the control group was given only gavage with 100 μL corn oil. Each mouse was given a gavage for 7 days a week. On the 14th day after exposure, half of the mice in each group were selected and sacrificed. The other half of the mice were kept and sacrificed on the 56th day after exposure. This study was approved by the Ethics Committee of Zhejiang University of Technology (No. 20200910117; approval date: 8 September 2020). 

### 2.2. Histopathological Analysis

After 56 days of DIF exposure, the colon samples in each group were collected. One part of the colon was cut off and put in 4% paraformaldehyde (Sangon, China) for 24 h. Then the colon samples were embedded in paraffin and cut into 5-μm-thick sections. Next, the sections were stained by alcin blue-periodic acid-schiff (AB-PAS). The immunohistochemical stain methods were similar to our previous study [18].

### 2.3. Colonic RNA Extraction and Real-Time qPCR

Total colonic RNA extraction was performed by Trizol (Vazyme, Nanjing, China) and then the RNA was quantified and revered by reverse transcriptase kit (Vazeyme, China). Further qPCR was performed with SYBR Green (Vazeyme, China) and Light Cycler 480 System II (Roche, Basel, Switzerland). The sequences of primers were the same as our previous studies [20]. The relative mRNA levels were corrected by the 2^−ΔΔCT^ method [21].

### 2.4. 16S rRNA Analysis

After exposure, the contents of colon in each mouse were collected, frozen in the liquid nitrogen and stored at −80 °C for further use. The total DNA in the colonic contents were extracted by Foreal Naotechnoloy magnetic bead DNA isolation kit (Hangzhou, China). The extraction strictly followed the manufacturer’s instructions. The changes at the phylum level after DIF exposure were examined by qPCR with the following steps: (1) 50 °C for 2 min, (2) 95 °C for 10 min, 95 °C for 15 s, 56 °C for 30 s and 72 °C for 1 min, repeated for 40 cycles, and (3) followed by 72 °C for 10 min. The primer sequences were the same as our previous studies [22,23]. 

The V3–V4 regions of 16S rRNA genes were amplified by PCR with 341F and 806R primers. Thermal cycling had the following conditions: (1) denaturation at 98 °C for 1 min, (2) denaturation at 98 °C for 1 min, annealing at 50 °C for 30 s and elongation at 72 °C for 30 s with 30 cycles, (3) 72 °C for 5 min. Then the PCR products were quantified and qualified. Next, the sequencing libraries were generated by TruSeq^®^ DNA PCR-Free Sample Preparation Kit (Illumina, San Diego, CA, USA) and sequenced by Illumina NovaSeq platform with 250 bp paired-end reads. The further bioinformatic analysis of raw data was performed by QIIME2 [24]. The database used to assign taxonomy was according to SILVA138. In addition, rarefaction curves of 16S rRNA sequencing in each group were shown in the Figure S1. The statistical test employed to assess significance at phylum and genus levels was student *t*-test.

### 2.5. Statistical Analysis

Bar graphs were expressed as Mean ± standard error of mean (SEM). The statistical significance was determined by one-way analysis of variance (ANOVA) with *p* < 0.05 and post hoc analysis was performed using Tukey’s multiple comparisons test. 

## 3. Results

### 3.1. Exposure to DIF Affected the Colonic Mucus Expression

**Figure 1:**
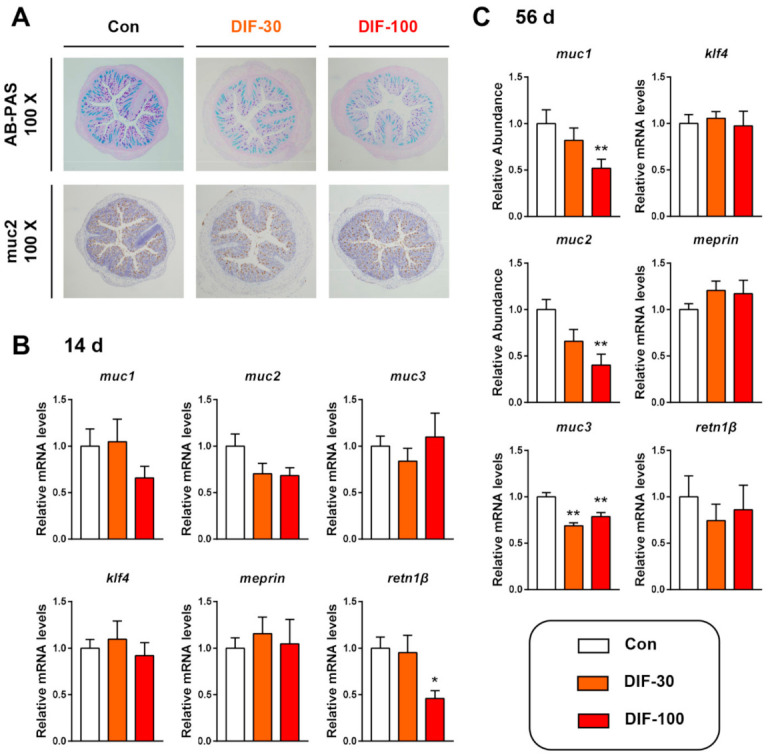
Effects of DIF exposure on the colonic mucus secretion in mice. (**A**) Histopathological analysis of colon by AB-PAS satin and immunochemical staining of *muc2* protein after 56 days of DIF exposure. The relative mRNA levels of mucin secretion related gens after (**B**) 14 days and (**C**) 56 days of DIF exposure. The presented data are the Mean ± SEM (*n* = 8). * *p* < 0.05, ** *p* < 0.01 versus control group.

The histopathological analysis showed that the colonic mucus expression decreased after 56 days of DIF exposure (Figure 1A). AB-PAS staining showed that 30 and 100 mg/kg/d DIF exposure for 56 days decreased the colonic mucus content, especially the mixture of acidic and neutral mucins (purple color) (Figure 1A). The immunochemical staining of mucin 2 (*muc2*) protein showed the decrease in *muc2* expression. Then, we examined the transcript levels of mucin secretion related genes in the colon of mice after the 14 and 56 days of DIF exposure. After 14 days of exposure, the mucin 1 (*muc1*) decreased in the DIF-100 group and *muc2* decreased in the DIF-30 and DIF-100 groups though without significance (Figure 1B). When compared with the control group, the relative mRNA levels of *retn1β* decreased significantly in the DIF-100 group (Figure 1B). After 56 days of exposure, the *muc1* and *muc2* decreased significantly in the DIF-100 groups and *muc3* decreased significantly in the DIF-30 and DIF-100 groups in comparison with the control group (Figure 1C). 

### 3.2. Exposure to DIF Affected the Gut Microbiota Composition

**Figure 2:**
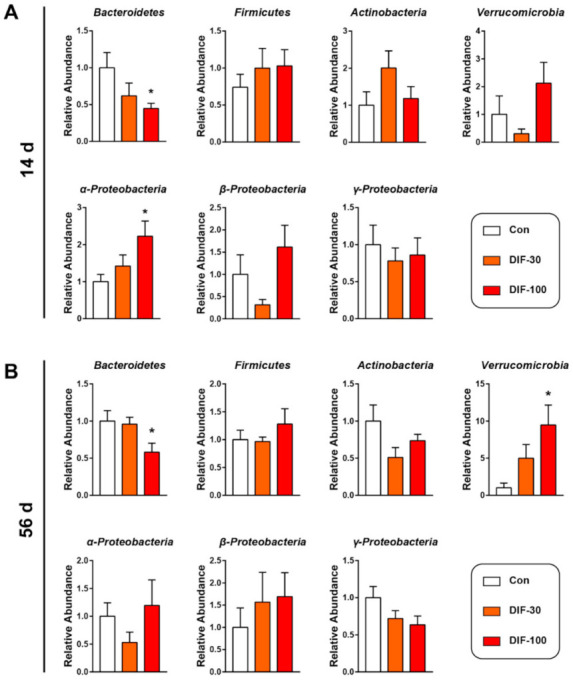
Effects of DIF exposure on the relative abundance of gut microbiota at phylum level in the gut of mice. The relative abundance of the gut microbiota at the phylum level after (**A**) 14 days and (**B**) 56 days of DIF exposure, including Bacteroidetes, Firmicutes, Actinobacteria, Verrucomicrobia, α-Proteobacteria, β-Proteobacteria and γ-Proteobacteria. The presented data are the Mean ± SEM (*n* = 8). * *p* < 0.05 versus control group.

In addition to the mucus layer, the gut microbiota also play a very important role in gut health [14]. Then, we investigated the gut microbiota composition at the phylum level after DIF exposure via RT-qPCR. After 14 days of exposure, the relative abundance of Bacteroidetes decreased significantly and α-proteobacteria increased significantly when compared with the control group (Figure 2A). The relative abundance of Verrucomicrobia and β-Proteobacteria decreased in the DIF-30 group and increased in the DIF-100 group, however, no significant difference was observed (Figure 1A). 

After 56 days of DIF exposure, the relative abundance of Bacteroidetes also decreased significantly in the DIF-100 group when compared with the control group (Figure 2B). The relative abundance of Verrucomicrobia increased dramatically in the DIF-100 group after 56 days of DIF exposure (Figure 2B). In addition, the relative abundance of Actinobacteria and γ-Proteobacteria tended to decrease after 56 days DIF exposure (Figure 2B). 

### 3.3. Data Analysis from 16S rRNA Sequencing

**Figure 3:**
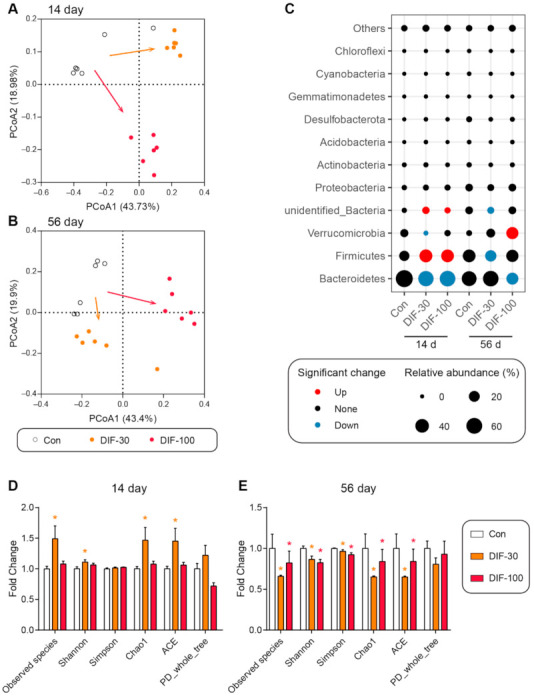
Effects of DIF exposure on the gut microbiota composition in the gut of mice. Principal coordinate analysis (PCoA) in gut microbiota after (**A**) 14 days and (**B**) 56 days of DIF exposure. (**C**) Relative abundance of gut microbiota at phylum level. The diversity indexes in the colonic gut microbiota after (**D**) 14 days and (**E**) 56 days of DIF exposure. * *p* < 0.05 versus control group.

The RT-qPCR results demonstrated that DIF exposure affected the gut microbiota composition. Then, the 16S rRNA sequencing was performed to further explore the changes of gut microbiota composition after DIF exposure. Firstly, the principal coordinate analysis (PCoA) showed that both 14 and 56 days of DIF exposure affected the gut microbiota composition in mice (Figure 3A,B). The DIF exposure caused different results between the DIF-30 and DIF-100 groups, whether after 14 or 56 days of exposure (Figure 3A,B). At the phylum level, the relative abundance of Bacteroidetes decreased significantly in the DIF-30 and DIF-100 groups after 14 days exposure (Figure 3C). The relative abundance of Verrucomicrobia decreased significantly in the DIF-30 group. Meanwhile, the relative abundance of *Firmicutes* and unidentified_Bacteria increased significantly in the DIF-30 and DIF-100 groups. As for the 56 days of DIF exposure, the relative abundance of *Firmicutes* and unidentified_Bacteria decreased significantly in the DIF-30 group (Figure 3C). Compared with the control group, the relative abundance of Bacteroidetes decreased significantly and Verrucomicrobia increased significantly in the DIF-100 group. 

In addition to the gut microbiota composition, some indexes of alpha diversity, including the observed species, Shannon, Chao1 and ACE, increased significantly in the DIF-30 group after 14 days of exposure (Figure 3D). As for 56 days of DIF exposure, the observed species, Shannon, Simpson, Chao1 and ACE, also decreased significantly in the DIF-30 and DIF-100 groups when compared with the control group (Figure 3E). 

### 3.4. Differential Genera after DIF Exposure

**Figure 4:**
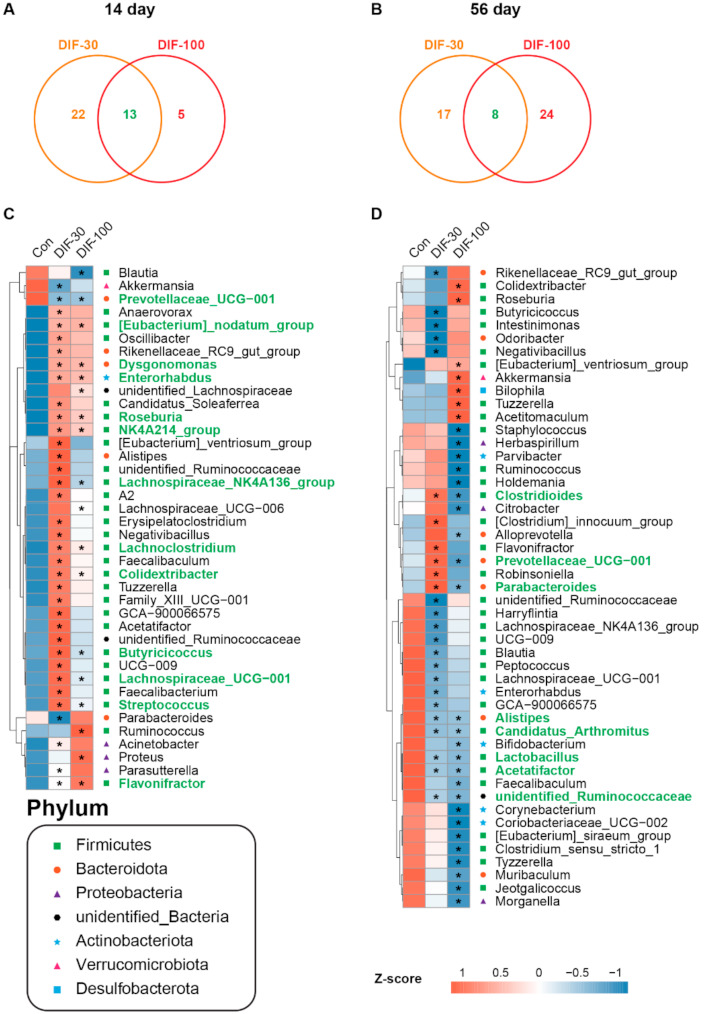
Effects of DIF exposure on the colonic gut microbiota at genus level in the gut of mice. Venn diagram of the differential genera between the DIF-30 and DIF-100 groups compared with the control group after (**A**) 14 days and (**B**) 56 days of DIF exposure. The heatmap of the differential genera after (**C**) 14 days and (**D**) 56 days of DIF exposure. * *p* < 0.05 versus control group.

Statistical analysis demonstrated that some genera were changed significantly after DIF exposure. There were 35 and 18 significantly changed genus in the DIF-30 and DIF-100 after 14 days of exposure, respectively (Figure 4). In the differential genus, there were 13 differential genera in two treated groups (Figure 4C). Compared with the control group, 14 days of DIF exposure significantly decreased the relative abundance of *Prevotellaceae_UCG-001* and increased the relative abundance of *[Eubacterium]-nodatum_group*, *Dysgonomonas*, *Enterorhabdus*, *Roseburia*, *NK4A214_group*, *Lachnospiraceae_NK4A136_group*, *Lachnoclostridium*, *Colidextribacter*, *Butyricicoccus*, *Lachnospiraceae_UCG-001*, *Streptococcus* and *Flavonifractor* (Figure 4C). 

After 56 days of DIF exposure, 25 and 32 significantly differential genera were observed in the DIF-30 and DIF-100 groups, respectively (Figure 4B). There were 8 differential genera between the DIF-30 and DIF-100 groups. The relative abundance of *Clostridioides*, *Prevotellaceae_UCG-001* and *Parabacteroides* increased significantly in the DIF-30 and decreased significantly in the DIF-100 group. In addition, the relative abundance of *Alistipes*, *Candidatus_Arthromitus*, *Lactobacillus*, *Acetatifactor* and *unidentified_Ruminococcaceae* decreased significantly in the DIF-30 and DIF-100 groups (Figure 4D).

## 4. Discussion

The use of DIF in agriculture to control fungi has a very long history. Due to the high efficiency and broad spectrum of DIF, it has been widely used worldwide. However, the excessive use of DIF caused high volumes of DIF residue in vegetables and fruits [2]. In addition, DIF was also detected in water bodies with high persistence [4,5,6]. The residue of DIF in water threaten the health of aquatic animals. For example, Zhu, et al. demonstrated that 1.2 mg/L DIF exposure induced cardiovascular toxicity in the larval zebrafish [7]. Jiang et al. also showed that 2 mg/L DIF exposure caused lipid metabolism disorder in the larval zebrafish [25]. In addition to the aquatic animals, our previous study also demonstrated that 100 mg/kg/d DIF exposure decreased the body weight, caused liver injury and caused lipid metabolism disorder in male mice [26]. These studies indicated that the health risk of DIF, which was considered to be a low toxicity fungicide, could not be ignored in animals.

Our previous studies suggested that xenobiotics, especially environmental pollutants, including antibiotics, microplastic and fungicides, caused gut microbiota disorder and impact the intestinal barrier [14,27,28]. The intestinal mucus layer is one crucial part of the gut barrier to preventing foreign invasion [16]. The mucins were synthesized, stored and secreted by goblet cells in the gut of animals [29]. When the mucus layer was defective, numerous bacteria could invade the host and cause serious immune response [17] and inflammatory bowel disease [30]. Here, the AB-PAS staining showed the decrease in colonic mucus after 56 days of DIF exposure (Figure 1A). Furthermore, the results of IHC and RT-qPCR also demonstrated the decrease in *muc2* after DIF exposure when compared with the control group (Figure 1A–C). Some similar results could also be found with other fungicides. For example, Jin, et al. showed that triazole fungicide imazalil exposure decreased the gut mucus secretion and induced inflammation in the liver and gut in the F0 and offspring [18]. *Muc2* was the major mucus protein that was highly O-glycosylated [31]. According to these results, it could be found that sub-chronic DIF exposure could influence the barrier function in mice.

The gut microbiota also play crucial roles in the host metabolism and immune response [32,33]. The composition of gut microbiota changed significantly when compared with the control group, both at 14 and 56 days of exposure (Figure 3A–C). Meanwhile, the alpha diversity was calculated to estimate the community richness and diversity. Some indexes of alpha diversity, including the observed species, Shannon, Chao1 and ACE, increased significantly in the DIF-30 group after 14 days of exposure (Figure 3D). The observed species, Shannon, Simpson, Chao1 and ACE decreased significantly in the DIF-30 and DIF-100 groups after 56 days of DIF exposure, indicating that sub-chronic DIF exposure decreased the gut microbiota diversity. At the genus level, we observed many differential genera after DIF exposure. For example, the relative abundance of *Dysgonomonas*, *Roseburia*, *Ruminococcus*, *Lachnoclostridium* and *Streptococcus* increased significantly after 14 days of exposure (Figure 4C). *Dysgonomonas* and *Butyricicoccus* were considered to be potential probiotic bacteria [34,35]. *Roseburia* and *Ruminococcus* could produce short chain fatty acids [36,37]. The short chain fatty acids were fermented by some gut microbiota and played an important role in gut health [38]. Some studies also showed that probiotic supplementation could mitigate the toxic effects of xenobiotics [39,40]. In addition, *Lachnoclostridium* was considered to be a pathogen-causing inflammatory disease [41,42] and *Streptococcus* caused infection in cirrhosis patients [43]. Gao et al. suggested that *Flavonifractor* was negatively correlated with obesity [44]. These results suggested that DIF exposure inducing microbiota dysbiosis might be associated with the metabolism change, however, the further mechanism still remained unclear.

Interestingly, after 56 days of DIF exposure, the relative abundance of *Alistipes*, *Acetatifactor* and *Lactobacillus* decreased significantly in the DIF-30 and DIF-100 groups (Figure 4D). *Alistipes* and *Acetatifactor* are butyrate-producing bacteria [45,46]. *Lactobacillus* was one major probiotic [47]. Interestingly, the relative abundance of *Akkermansia* increased significantly after 100 mg/kg/d DIF exposure for 56 days. As we know, *Akkermansia* was firstly isolated from healthy adult feces and uses mucin as the sole carbon source [48]. Some studies demonstrated that *Akkermansia* was one potential probiotic in short chain fatty acids and branched-chain fatty acids production, improving obesity [49,50,51], diabetes [52] and inflammation [53,54]. Recently, some studies challenged this view and demonstrated that the higher abundance of *Akkermansia* might aggravate colitis [55,56]. According to our previous study, as a chiral fungicide, DIF had two chiral centers consists of four stereoisomers, (2R,4S), (2R,4R), (2S,4S) and (2S,4R). Our previous study showed that different stereoisomer caused different changes in the gut microbiota and the (2R,4S)-DIF exposure caused the most significant decrease in the diversity of gut microbiota with the blooming of the *Akkermansia* [57]. However, the probiotic effects of 100 mg/kg BW/d DIF that induced the increase in *Akkermansia* needed further discussion.

In this study, 7-week-old male mice were exposed to 30 and 100 mg/kg BW/d DIF for 14 and 56 days. The histopathological analysis and qPCR demonstrated the decrease in *muc2* after 56 days of DIF exposure. The further 16S rRNA sequencing showed that the gut microbiota composition was significantly changed after DIF exposure and several differential genera were also found. In conclusion, sub-chronic DIF exposure impact colonic mucus expression and caused gut microbiota dysbiosis. These results are a reminder that the hazardous effects of DIF on gut health should not be ignored.

## Supplementary Materials

The following supporting information can be downloaded at: https://www.mdpi.com/article/10.3390/toxics10010034/s1, Figure S1: Rarefaction curves of 16S rRNA sequencing after 14 days of DIF exposure and 56 days of DIF exposure.
