# Supplementary material for: Sub-Chronic Difenoconazole Exposure Induced Gut Microbiota Dysbiosis in Mice
Source: Toxics. 2022 Jan 12;10(1):34. doi: 10.3390/toxics10010034 (PMC8780654; doi:10.3390/toxics10010034)
Supplement: Supplementary file 1 [file toxics-10-00034-s001.zip › toxics-1507632 -proof supplementary.pdf]

toxics

MDPI

Supplementary Materials: Sub-chronic Difenoconazole Exposure Induced Gut Microbiota Dysbiosis in Mice

Zhiwei Bao, Weitao Wang, Xiaofang Wang, Mingrong Qian and Yuanxiang Jin

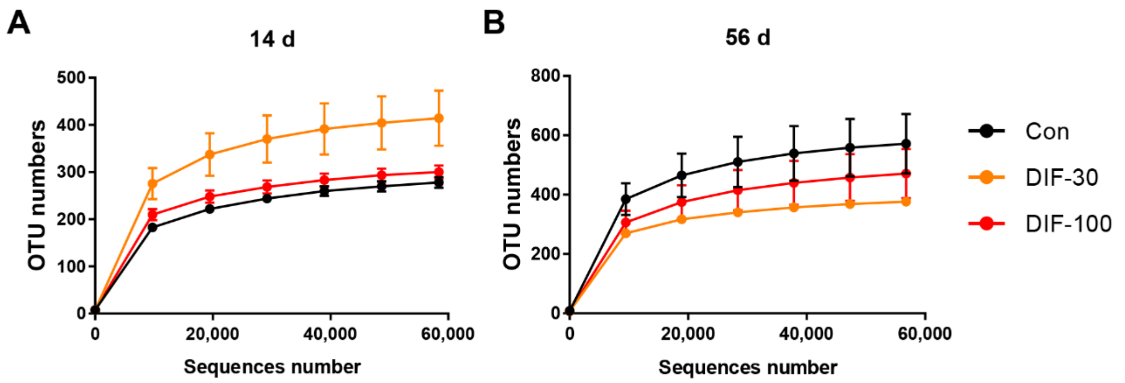

Figure S1. Rarefaction curves of 16S rRNA sequencing after (**A**) 14 days of DIF exposure and (**B**) 56 days of DIF exposure. The presented data are the Mean ± SEM (*n* = 6).
